# Revolutionization of the Leadless Pacemaker Implantation: Hemodynamic Verification Slashes Vascular Complications

**DOI:** 10.31083/RCM43879

**Published:** 2026-02-11

**Authors:** Xin Zhang, Lingyun Jiang, Yuanning Xu, Xingbin Liu

**Affiliations:** ^1^Department of Cardiology, West China Hospital of Sichuan University, 610041 Chengdu, Sichuan, China

**Keywords:** leadless pacemaker, Micra, vascular complications, femoral access, hemodynamic verification

## Abstract

**Background:**

The procedure of implanting a Micra leadless pacemaker (Medtronic PLC, Dublin, Ireland) via transfemoral venous access carries the risk of vascular complications. Our study examined whether Liu's hemodynamic verification technique, a basic bedside evaluation of flow and pulsatility through the sheath side port before dilator advancement, minimizes vascular complications in Micra implantation.

**Methods:**

We conducted a retrospective analysis of 465 consecutive Micra implantations performed at the Department of Cardiology, West China Hospital of Sichuan University, from December 2019 to November 2023. Participants were categorized into two groups: Group A (n = 389), which employed pre-dilation hemodynamic verification with sheath blood flow analysis (Liu's method), and Group B (n = 76), which used standard vascular access. The groups were compared based on demographics, procedural specifics, and vascular complications.

**Results:**

Compared with the standard puncture method, Liu's technique was linked to a much lower incidence of vascular complications (0.5% vs. 3.9%; *p* < 0.05). No major vascular complications necessitating surgical or endovascular treatment occurred in Group A (0% vs. 2.63%; *p* < 0.01). The method allowed quick identification of accidental arterial entry and immediate corrective actions without requiring extra specialized tools. No increase in procedure duration or complications related to the Micra device was observed.

**Conclusion:**

In this single-center retrospective study of 465 consecutive Micra implantations, Liu's method for hemodynamic verification significantly reduced the rate of vascular complications and completely prevented major vascular events compared with traditional femoral venipuncture. The technique is straightforward, economical, easy to learn, and could be a viable option when ultrasound guidance is not accessible.

## 1. Introduction

Pacemakers are commonly employed to treat slow heart rhythms, such as sinus node issues and atrioventricular conduction problems [1]. Despite ongoing advancements in technology, the complication rate for conventional transvenous pacemaker implantation remains between 3.8% and 12.4% [2, 3]. These complications, such as pneumothorax, cardiac perforation, pocket hematoma, and lead-related problems, can greatly affect patient outcomes and healthcare expenses [4].

In 2016, the US Food and Drug Administration approved leadless pacemakers to overcome the shortcomings of traditional pacemaker systems [5]. Using a 27 French hydrophilic-coated introducer sheath, the Micra transcatheter pacing system is placed percutaneously through the femoral vein into the right ventricle [5]. Large-scale registry studies have indicated high rates of successful implantation, with a significant decrease in long-term complications and the need for pacemaker revisions compared to conventional systems [6, 7, 8].

Nonetheless, complications related to vascular access, especially arteriovenous fistulas and pseudoaneurysms, with an incidence rate of about 1.2% [9]. A study found that using real-time ultrasound guidance and the Z-suture technique during leadless pacemaker implantation reduces vascular complications [9]. Vascular closure devices like the double Perclose ProGlide aid hemostasis, lower venous thrombosis risk, improve patient comfort, and allow early ambulation [10]. However, employing these methods necessitates expert skills and the presence of the right equipment. In clinical settings lacking these resources, its application is significantly limited, reducing its overall usefulness.

In response to these challenges, we have developed an innovative vascular examination technique that employs the assessment of blood flow dynamics through the delivery sheath to identify and prevent inadvertent arterial puncture before vessel dilation. This study seeks to evaluate the effectiveness of this hemodynamic verification method in minimizing vascular complications associated with Micra implantation procedures, without necessitating additional specialized equipment or extensive operator training.

## 2. Materials and Methods

### 2.1 Study Population and Data Collection

This single-center, retrospective cohort study included 465 consecutive patients who underwent Micra Transcatheter Leadless Pacing System (Medtronic plc, Dublin, Ireland) implantation at the Department of Cardiology, West China Hospital of Sichuan University from December 2019 to November 2023. The study protocol was approved by the Ethics Committee of West China Hospital, Sichuan University (Approval No. 2019 Review (1079)) in accordance with the Declaration of Helsinki.

Baseline patient characteristics (including demographics, clinical diagnoses, comorbidities, and medication regimens) and procedure-related data were extracted from electronic medical records. Follow-up information was obtained through outpatient clinic visits and and phone consultations.

This retrospective study did not include an a priori samplesize calculation because it analyzed all consecutive Micra implantations performed at our center during the study period. Thus, the study is a real-world, single-center consecutive case series instead of a trial with prospective power.

### 2.2 Implantation Procedure

Leadless pacemaker implantation procedures followed standard techniques as described in previous literature [11]. All procedures were performed under local anesthesia via femoral venous access. Following standard sterile preparation and draping, femoral venous puncture was performed using the Seldinger technique under anatomical guidance. Following implantation, hemostasis was attained by applying manual compression for at least 5 minutes, followed by a compression dressing after removing the sheath. Observations were made on patients for any local bleeding and formation of hematomas.

#### Liu’s Vascular Examination Method

To reduce vascular complications during puncture procedures, Professor Liu Xingbin from West China Hospital of Sichuan University developed an innovative vascular examination technique, which we refer to as Liu’s vascular examination method.

**Fig. 1.**
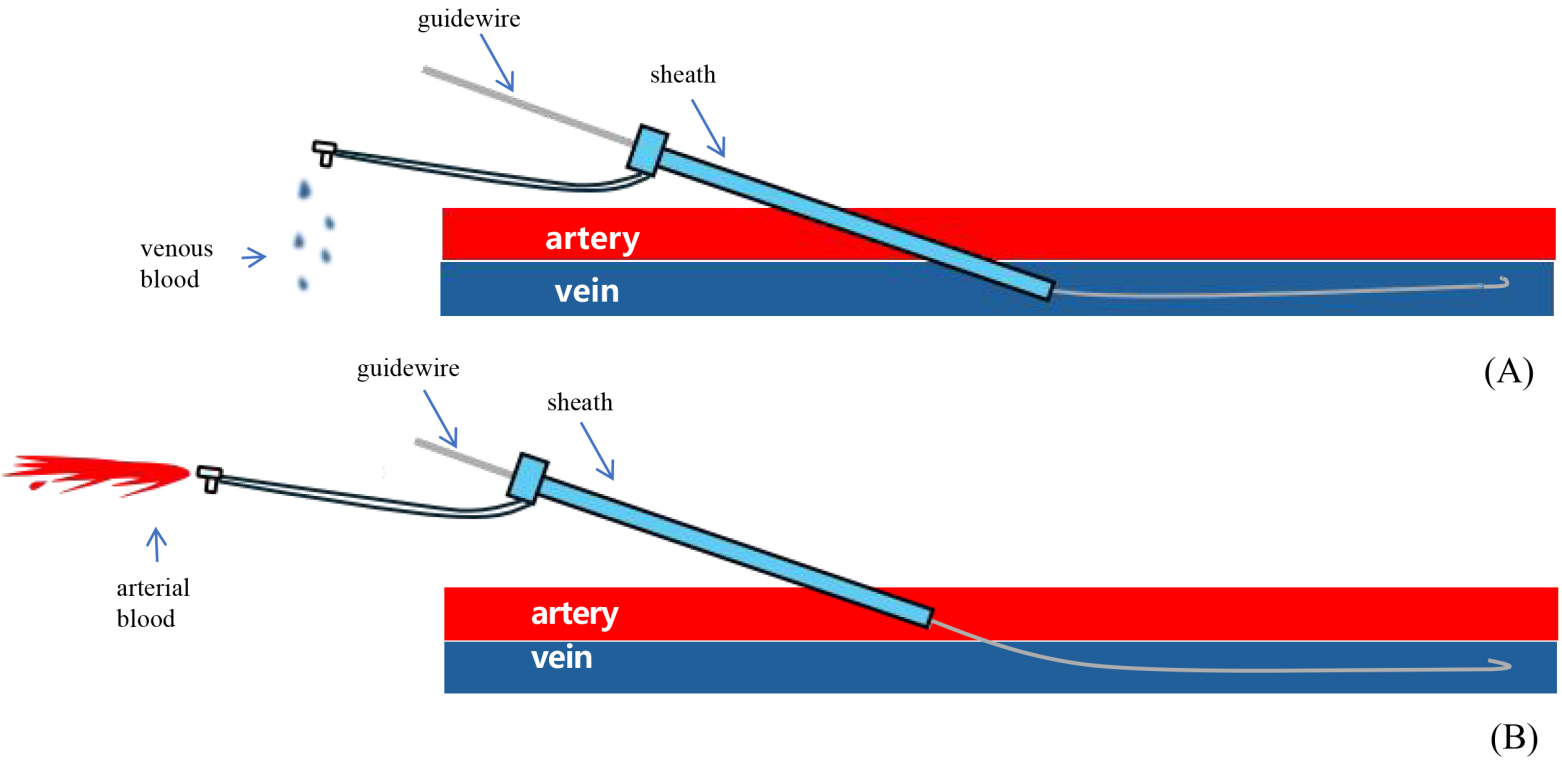
**Liu’s vascular examination method: maintain the position of the guidewire while gradually withdrawing the sheath**. (A) The sheath punctured the vein without damaging the artery: the velocity of venous blood flow is reduced, resulting in a dark red coloration. (B) The sheath has traversed the arterial vessel and penetrated the vein: arterial blood flow is characterized by rapid velocity and a vibrant crimson coloration.

The procedure for Liu’s vascular examination method was as follows: After successful puncture, a 5F delivery sheath (Cordis, Johnson, USA) was inserted into the femoral vein under guidewire guidance. After removing the inner dilator, the position of the guidewire was maintained while the outer sheath was gradually retracted. During this process, the velocity and color of blood flow from the sheath’s side port were closely monitored. If blood flow velocity suddenly increased or appeared bright red, it indicated that the sheath had traversed an arterial vessel and entered the vein, necessitating immediate re-puncture. If bright red blood extrusion was observed (Fig. 1), it suggested femoral artery entry, requiring immediate guidewire removal and pressure hemostasis. This technique effectively reduced the risk of vascular complications, including bleeding, arteriovenous fistula, and pseudoaneurysm formation (**Supplementary Material Videos**).

### 2.3 Study Groups

Patients were divided into two groups based on whether Liu’s vascular examination method was employed during the procedure:

Group A (n = 389): Patients who underwent hemodynamic verification using Liu’s vascular examination method prior to vessel dilation with expandable sheaths.

Group B (n = 76): Patients who underwent conventional venous access without this additional verification step provided by Liu’s method.

In Group A, Liu’s method for vascular examination was conducted following a successful puncture but prior to the dilation of the vessel using expandable sheaths, while patients in Group B did not undergo this examination. In both groups, heparinized saline was continuously used to flush the side ports of the introducer and delivery system to minimize clot formation. Antiplatelet treatments continued without interruption during the study, but anticoagulation was halted 24 hours prior to the procedure, unless the patient was deemed at high risk for thromboembolism.

### 2.4 Outcome Measures

The main endpoint was the occurrence of any vascular complications, and the secondary endpoints included major complications that necessitated intervention. Prior studies defined major complications as events leading to death, hospitalization, prolonged hospital stays, or device malfunctions [12]. The vascular issues included groin hematoma, bleeding in the retroperitoneal area, pseudoaneurysm, arteriovenous fistula, and the requirement for blood transfusions, vascular procedures, or surgeries.

Technical and electrical parameters, such as sensing amplitude, pacing threshold, and impedance, were documented and assessed at the time of implantation and during follow-up after the procedure. All patients underwent systematic follow-up to assess device functionality and the occurrence of complications.

#### Statistical Analyses

For continuous variables that follow a normal distribution, data are shown as the mean ± SD, while categorical variables are presented as frequencies (%). A multivariate Cox proportional hazards regression model was used to assess the link between antiplatelet/anticoagulation therapy and vascular complications in each group, estimating odds ratios (ORs) and 95% confidence intervals. A 2-tailed *p* < 0.05 was considered significant in all analysis. All analyses were performed using Empower (R) (http://www.empowerstats.com, X&Y solutions, Inc., Boston MA) and R (http://www.R-project.org).

## 3. Results

### 3.1 Patient Characteristics

Between December 2019 and November 2023, 465 patients consecutively underwent the implantation of the Micra transcatheter leadless pacemaker. In the study, the subjects were categorized into two groups: Group A, with 389 members, where Liu’s method for vascular examination was used before vessel dilation, and Group B, with 76 members, where the traditional approach without hemodynamic verification was applied.

The baseline characteristics of the two groups are summarized in Table 1. Group A had 211 males, accounting for 54% of the group, with an average age of 74 ± 15 years. Group B consisted of 41 males, also 54% of the group, with an average age of 72 ± 19 years. The prevalence of comorbidities was similar between groups, with diabetes mellitus (30% vs. 31%), atrial fibrillation (29% vs. 30%), chronic renal failure (22% vs. 20%), and chronic pulmonary disease (14% vs. 14%) being the most common in Groups A and B, respectively. The main difference in baseline characteristics was that more patients in Group B were on antiplatelet medications compared to Group A (18% vs. 8%, *p* < 0.05).

**Table 1.** **Patient characteristics at admission**.

N	Group A	Group B	*p*-value
	389	76	
Age (years)	74.43 ± 15.31	72.12 ± 19.42	0.588
Male sex	211 (54.24%)	41 (53.95%)	0.962
Body mass index (kg/m^2^)	23.59 ± 3.88	23.44 ± 3.51	0.784
LVEF (%)	66.46 ± 6.62	67.17 ± 6.17	0.276
Hypertension	49 (64.47%)	223 (57.47%)	0.257
Atrial fibrillation	114 (29.31%)	23 (30.26%)	0.867
Myocardial infraction	7 (1.80%)	0 (0.00%)	0.605
Diabetes mellitus	115 (29.56%)	24 (31.58%)	0.725
Stroke	19 (4.90%)	7 (9.21%)	0.135
Chronic renal failure	86 (22.11%)	15 (19.74%)	0.647
Hemodialysis	13 (3.35%)	4 (5.26%)	0.417
Chronic pulmonary disease	53 (13.62%)	11 (14.47%)	0.844
Cancer	7 (1.80%)	0 (0.00%)	0.605
Pacemaker reimplant after pocket infection	13 (3.34%)	5 (6.58%)	0.181
Alzheimer’s disease	11 (2.84%)	0 (0.00%)	0.137
Lack of upper vascular extremity access	6 (1.54%)	2 (2.63%)	0.623
Anticoagulation agent	51 (13.11%)	16 (21.05%)	0.071
Antiplatelet agent	32 (8.23%)	14 (18.42%)	0.006

Data are expressed as mean ± standard deviation or number (%). Group A: patients who underwent hemodynamic verification using Liu’s vascular examination method prior to vessel dilation with expandable sheaths; Group B: patients who underwent conventional venous access without this additional verification step provided by Liu’s method; LVEF, left ventricular ejective fraction.

### 3.2 Implantation Characteristics

According to Table 2, atrioventricular block was the leading reason for pacing in both Group A (62%) and Group B (58%, *p* = 0.47). In Group A, 374 patients (96.1%) had successful device deployment on the first attempt, while in Group B, 73 patients (96.1%) experienced the same. Using Liu’s vascular examination technique significantly lowered the rate of vascular complications post-Micra implantation. In Group A, the rate of vascular complications was 0.5% (2 out of 389), whereas in Group B, it was 3.9% (3 out of 76), with a statistically significant difference (*p* < 0.05).

**Table 2.** **Implantation characteristics and complications**.

N		Group A	Group B	*p*-value
		389	76	
Pacing indication				
	Atrioventricular block	243 (62.47%)	44 (57.89%)	0.453
	Sick sinus syndrome	160 (41.13%)	38 (50.00%)	0.153
Procedure with two or more positioning attempts		15 (3.86%)	3 (3.95%)	0.970
Vascular complications		2 (0.51%)	3 (3.95%)	0.008

Data are expressed as mean ± standard deviation or number (%).

In Group A, Liu’s method for examining blood vessels detected two instances of accidental arterial puncture prior to vessel dilation using expandable sheaths. In both situations, the guidewire was quickly removed, and pressure was applied to the puncture location, resulting in a successful resolution with no lasting effects. On the third day after the procedure, these patients were discharged without any signs of arteriovenous fistula or pseudoaneurysm.

On the other hand, Group B encountered three major vascular complications at the puncture site. Surgical intervention was needed for two patients (2.63%), while vascular embolization was required for one patient (1.31%). Significantly, there were no major vascular complications in Group A that required surgical or interventional actions (0% vs. 2.63%, *p* < 0.01).

### 3.3 Electrical Characteristics

Table 3 shows the electrical properties during the implantation and follow-up phases. During the implantation procedure, patients in Group A (n = 389) exhibited significantly higher pacing thresholds compared to Group B patients (n = 76) (0.64 ± 0.35 V vs. 0.51 ± 0.27 V, *p* < 0.05). At the same time, Group A demonstrated significantly lower pacing impedance than Group B (839.63 ± 255.99 Ω vs. 910.87 ± 314.04 Ω, *p* < 0.05). However, no significant difference was observed in R wave amplitude between the two groups (9.32 ± 4.74 mV vs. 9.86 ± 4.33 mV, *p* = 0.24).

**Table 3.** **Electrical features at implant and during follow-up**.

	Implant procedure			Follow-up		
	Group A	Group B	*p*-value	Group A	Group B	*p*-value
	(n = 389)	(n = 76)		(n = 389)	(n = 76)	
Pacing threshold, V/0.24 ms	0.64 ± 0.35	0.51 ± 0.27	0.003	0.58 ± 0.39	0.59 ± 0.47	0.921
Pacing impedance, Ω	839.63 ± 255.99	910.87 ± 314.04	0.035	609.73 ± 146.79	598.06 ± 115.63	0.776
R wave amplitude, mV	9.32 ± 4.74	9.86 ± 4.33	0.236	11.63 ± 4.00	12.90 ± 4.48	0.030
Follow-up duration (days)				299.90 ± 257.46	592.61 ± 396.44	<0.001

During the follow-up period, both groups showed comparable pacing thresholds (0.58 ± 0.39 V vs. 0.59 ± 0.47 V, *p* = 0.92) and pacing impedance (609.73 ± 146.79 Ω vs. 598.06 ± 115.63 Ω, *p* = 0.78) with no statistically significant differences. Notably, patients in Group B demonstrated significantly higher R wave amplitudes compared to Group A (12.90 ± 4.48 mV vs. 11.63 ± 4.00 mV, *p* = 0.03). Patients in Group B were observed for a notably longer time (592.61 ± 396.44 days) than those in Group A (299.90 ± 257.46 days), with a significant difference (*p* < 0.05).

### 3.4 A Multivariate Cox Regression Analysis

Table 4 provides stratified estimates of the link between antiplatelet and anticoagulation therapies and the incidence of vascular complications, detailed separately for Group A and Group B. According to the stratified analyses in Table 4, there was no statistically significant connection between antiplatelet or anticoagulation therapy and vascular complications in Group A or Group B. The relationship between group assignment and vascular complications did not significantly change after adjusting for sex, age, and body mass index (BMI).

**Table 4.** **A multivariate Cox regression analysis examined the link between antiplatelet/anticoagulation therapy and vascular complications in each group**.

Subgroups	Group A				Subgroups	Group B			
	OR (95% CI)	*p* value	Adjusted OR (95% CI)	*p* value		OR (95% CI)	*p* value	Adjusted OR (95% CI)	*p* value
Antiplatelet	0.000 (0.000, Inf)	0.100	0.000 (0.000, Inf)	0.100	Antiplatelet	1.994 (0.181, 22.001)	0.573	2.281 (0.164, 31.823)	0.540
Anticogulation	2.151 (0.124, 37.232)	0.609	1.330 (0.039, 44.773)	0.874	Anticiogulation	0.000 (0.000, Inf)	0.100	0.000 (0.000, Inf)	0.100

Adjust for: Sex; Age; body mass index (BMI). OR, odds ratio; CI, confidence interval.

## 4. Discussion

The present study demonstrates that Liu’s vascular examination method significantly reduces vascular complications associated with leadless pacemaker implantation (0.51% in Group A vs. 3.95% in Group B, *p* < 0.05). Notably, no major vascular complications requiring surgical intervention were observed in the group utilizing this technique (Group A), while 2.63% of patients in the control group (Group B) required vascular surgical or embolization interventions.

Leadless pacemakers represent a major advancement in cardiac pacing, offering a promising alternative to the conventional transvenous systems. Leadless pacemakers, although beneficial, can experience complications, particularly in the short term after being implanted [13]. Vascular complications, which may arise within the first month after implantation, are a significant concern [13]. The use of large delivery sheaths in leadless pacemaker implantation is a key factor in vascular complications, often resulting in notable vascular access challenges [14]. An investigation into the effectiveness of real-time ultrasound guidance combined with the Z-suture technique revealed a reduction in total and major vascular complications during leadless pacemaker implantation [9]. This research emphasizes the significance of using advanced procedural methods to reduce vascular damage and enhance patient results. Moreover, employing vascular closure devices like the double Perclose ProGlide has proven effective in promoting hemostasis and lowering the risk of venous thrombosis, which improves patient comfort and allows for early mobility [10]. Reports of individual cases demonstrate the risk of vascular complications post leadless pacemaker implantation. For example, a patient developed an arteriovenous fistula because of an abnormality in the deep femoral artery, highlighting the importance of thorough vascular evaluation before device implantation [15]. By accurately identifying arteries and monitoring dynamic flow, Liu’s vascular examination method offers a mechanical benefit over conventional anatomical landmark methods, effectively closing the safety gap between blind puncture and image-guided procedures.

This method, which serves as an alternative to ultrasound, provides substantial economic advantages, particularly in environments with limited resources. Conventional ultrasound equipment demands a large capital outlay and ongoing maintenance costs each year. Liu’s technique reduces costs and provides better safety results than traditional fluoroscopy-guided methods. The lack of significant vascular complications in the 389 patients treated with Liu’s method (Group A) probably led to substantial cost reductions for the healthcare system, especially when taking into account that significant expenses are incurred in the management of vascular complications [16, 17]. Liu’s method, unlike ultrasound-guided techniques, does not incur additional equipment or maintenance expenses, as a result, significantly lowering procedural expenses and financial barriers to its widespread use, especially in healthcare environments with resource limitations.

One reason Liu’s vascular examination method is effective is its straightforward learning curve. Studies indicate that operators typically need to complete 20–25 supervised cases to consistently perform ultrasound-guided venipuncture proficiently [18, 19]. In contrast, operators quickly adopted Liu’s method after just 1–2 supervised cases, demonstrating a rapid learning curve.

The application of antiplatelet and anticoagulation therapies in patients having leadless pacemaker implants is controversial due to the possible rise in vascular complications. A study found that patients with chronic kidney disease and end-stage renal disease who received leadless pacemakers had higher rates of major complications, including peripheral vascular issues, potentially worsened by anticoagulation therapy [20]. Moreover, coagulopathy was found to be a major predictor of procedural complications in patients receiving leadless pacemaker implants, highlighting the increased risk associated with anticoagulation [21]. Although there are risks, some studies propose that under particular conditions, it could be safe and feasible to continue anticoagulation therapy during the implantation of leadless pacemakers. A study on the Micra leadless pacemaker showed that continuous anticoagulation did not significantly raise the risk of major complications, such as access site issues and pericardial effusion, compared to those not receiving anticoagulation therapy [22]. Supporting this finding, another study indicated that patients with the Micra transcatheter pacing system experienced low rates of bleeding and thromboembolic complications, independent of their anticoagulation status [23]. Our study found no statistically significant association between antiplatelet or anticoagulation therapy and vascular complications in either group. These findings indicate that by carefully selecting patients and managing procedures, the risks linked to antiplatelet or anticoagulation therapy during leadless pacemaker implantation can be reduced.

The prevention of major vascular complications in our study is due to three key advantages of Liu’s method: real-time detection of arterial puncture through a 200% increase in pulsatile flow velocity during sheath retraction, 100% specificity of bright red blood in the 5F sheath indicating femoral artery entry, and an early intervention protocol that removes the guidewire and applies compression upon arterial entry detection.

The main limitation of this study is its retrospective observational design, which restricts the ability to definitively determine causality. The absence of randomization may result in selection bias and residual confounding factors. However, this research offers an understanding of real-life experiences with vascular issues linked to Micra implantation at a tertiary cardiac facility within a universal healthcare framework. To further validate Liu’s vascular examination technique, prospective multicenter randomized controlled trials are required.

## 5. Conclusion

In summary, our study over five years of Micra implantation indicates that Liu’s vascular examination technique effectively minimizes vascular complications related to leadless pacemaker implantation. This method is simple, requires little extra training, doesn’t need special equipment, and provides substantial clinical and economic advantages, making it an ideal alternative to ultrasound guidance when advanced imaging is not available.

## Availability of Data and Materials

The data that support the findings of this study are available from the corresponding author upon reasonable request.

## Supplementary Material

Supplementary material associated with this article can be found, in the online version, at https://doi.org/10.31083/RCM43879.
